# Time Course-Dependent Methanogenic Crude Oil Biodegradation: Dynamics of Fumarate Addition Metabolites, Biodegradative Genes, and Microbial Community Composition

**DOI:** 10.3389/fmicb.2017.02610

**Published:** 2018-01-04

**Authors:** Courtney R. A. Toth, Lisa M. Gieg

**Affiliations:** Petroleum Microbiology Research Group, Department of Biological Sciences, University of Calgary, Calgary, AB, Canada

**Keywords:** crude oil, methanogenesis, biodegradation, fumarate addition, anaerobic, hydrocarbon, heavy oil formation

## Abstract

Biodegradation of crude oil in subsurface petroleum reservoirs has adversely impacted most of the world's oil, converting this resource to heavier forms that are of lower quality and more challenging to recover. Oil degradation in deep reservoir environments has been attributed to methanogenesis over geological time, yet our understanding of the processes and organisms mediating oil transformation in the absence of electron acceptors remains incomplete. Here, we sought to identify hydrocarbon activation mechanisms and reservoir-associated microorganisms that may have helped shape the formation of biodegraded oil by incubating oilfield produced water in the presence of light (°API = 32) or heavy crude oil (°API = 16). Over the course of 17 months, we conducted routine analytical (GC, GC-MS) and molecular (PCR/qPCR of *assA* and *bssA* genes, 16S rRNA gene sequencing) surveys to assess microbial community composition and activity changes over time. Over the incubation period, we detected the formation of transient hydrocarbon metabolites indicative of alkane and alkylbenzene addition to fumarate, corresponding with increases in methane production and fumarate addition gene abundance. Chemical and gene-based evidence of hydrocarbon biodegradation under methanogenic conditions was supported by the enrichment of hydrocarbon fermenters known to catalyze fumarate addition reactions (e.g., *Desulfotomaculum, Smithella*), along with syntrophic bacteria (*Syntrophus*), methanogenic archaea, and several candidate phyla (e.g., “Atribacteria”, “Cloacimonetes”). Our results reveal that fumarate addition is a possible mechanism for catalyzing the methanogenic biodegradation of susceptible saturates and aromatic hydrocarbons in crude oil, and we propose the roles of community members and candidate phyla in our cultures that may be involved in hydrocarbon transformation to methane in crude oil systems.

## Introduction

Methanogenic hydrocarbon degradation is the leading model to explain the widespread occurrence of biodegraded oils and gas formation in oxidant-free reservoirs, whereby lighter oil components (e.g., saturates and aromatic hydrocarbons) are transformed to heavier forms that are of lower quality and more challenging to recover (Head et al., 2003, 2014; Larter et al., 2008). In microbiologically-active reservoirs (<80°C), biogeochemical estimates of hydrocarbon degradation to methane average on the order of 10^−3^-10^−4^ kg/m^2^/year at the oil-water interface (Head et al., 2003; Larter et al., 2006), impacting close to 840,000 barrels of crude oil annually^1^ Though most studies have focused on the degradation of single model hydrocarbon substrates (reviewed by Foght, 2008; Rabus et al., 2016), the number of reports detailing the susceptibility of whole crude oil to methanogenic biodegradation has surged in recent years (e.g., Townsend et al., 2003; Jones et al., 2008; Feisthauer et al., 2010; Gieg et al., 2010; Gray et al., 2011; Siegert et al., 2011; Mbadinga et al., 2012; Aitken et al., 2013; Sherry et al., 2013, 2014; Tan et al., 2013; Berdugo-Clavijo and Gieg, 2014; Bian et al., 2015; Cai et al., 2015; Liang et al., 2015; Xia et al., 2016). In particular, linear alkanes (e.g., Zengler et al., 1999; Anderson and Lovley, 2000; Gray et al., 2011; Liang et al., 2015; Tan et al., 2015b) and alkyl-substituted aromatics (e.g., Grbić-Galić and Vogel, 1987; Edwards and Grbić-Galić, 1994; Beller and Edwards, 2000; Washer and Edwards, 2007; Berdugo-Clavijo et al., 2012; Fowler et al., 2012, 2014) have been shown to be the most readily biodegradable hydrocarbon molecules in crude oil and in other fuel mixtures under methanogenic conditions.

Research in the past two decades has demonstrated fumarate addition as a possible anaerobic activation mechanism for *n*-alkanes and alkyl-substituted aromatics (Beller and Spormann, 1997; Annweiler et al., 2000; Beller and Edwards, 2000; Kropp et al., 2000; Rabus et al., 2001; Kniemeyer et al., 2003; Rios-Hernandez et al., 2003; Wilkes et al., 2003; Cravo-Laureau et al., 2005; Davidova et al., 2005). While this mechanism of hydrocarbon activation has been characterized using isolates, fumarate addition has also been shown to occur in mixed anaerobic hydrocarbon-degrading cultures (Foght, 2008; Rabus et al., 2016). Genes for fumarate addition enzymes (e.g., *ass*/*mas* encoding alkylsuccinate synthase for alkanes or *bss* encoding benzylsuccinate synthase for alkylbenzenes) have been detected in numerous methanogenic oil-degrading enrichment cultures (e.g., Zhou et al., 2012; Aitken et al., 2013; Tan et al., 2013; Berdugo-Clavijo and Gieg, 2014) and in hydrocarbon-containing environments (e.g., Callaghan et al., 2010; An et al., 2013; von Netzer et al., 2013; Johnson et al., 2015; Stagars et al., 2016), but their overall importance to the biotransformation of crude oil *in situ* is not clear. Recent investigations have begun to assess the prevalence of anaerobic hydrocarbon-degrading mechanisms including fumarate addition in methanogenic crude oil systems by combining metabolite profiling and targeted functional gene analysis approaches. In one example, Aitken et al. (2013) performed a 686-day time-course experiment comparing the degradation of crude oil alkanes under sulfate-reducing and methanogenic conditions. While the authors detected an accumulation of (1-methylalkyl) succinates over time in sulfate-reducing cultures (corresponding with an increase in *assA*/*masD* gene abundance), no such evidence was observed in parallel methanogenic replicates (relative to controls). This led Aitken et al. (2013) to postulate that an alternate pathway may be responsible for alkane activation under methanogenic conditions. A similar proposal was also made for a denitrifying *n*-hexadecane-degrading enrichment culture and by certain strains of sulfate-reducing bacteria such as Hxd3 (Callaghan et al., 2006, 2009). In contrast, Bian et al. (2015) obtained extensive metabolic and functional evidence of fumarate addition to alkanes from production fluids collected from three methanogenic oil fields, coinciding with the recovery of more than a dozen unique *assA*/*masD* gene sequences. Other putative hydrocarbon activation mechanisms include carboxylation, hydroxylation, or methylation, all of which have been reported to occur under other anaerobic electron-accepting conditions (Foght, 2008; Widdel et al., 2010).

Overall, a better understanding of the mechanisms and microbial consortia catalyzing the methanogenic attack of susceptible crude oil components is needed to help understand the metabolic processes governing heavy oil formation in petroleum reservoirs. Time course metabolic experiments, such as those conducted by Aitken et al. (2013), can help offer valuable insight into characterizing potentially transient microbial processes catalyzing crude oil biodegradation (more so than in single time-point experiments). Our research group previously demonstrated that the conversion of crude oil to methane was possible using a produced water consortium from a heavy oil field as an inoculum; single-time point chemical and *assA* gene analysis suggested fumarate addition as a possible mechanism of alkane activation (Berdugo-Clavijo and Gieg, 2014). In the present study, we build upon this preliminary observation by examining methanogenic crude oil biodegradation in a time course-dependent manner (using produced water from the same oil field) to better understand the chemical, functional, and microbial community dynamics of oilfield consortia involved in this metabolic process. Based on widespread evidence that fumarate addition catalyzes the activation of model hydrocarbon substrates (e.g., saturates, aromatics) under all anoxic conditions, we hypothesized that fumarate addition is a prevalent microbial process used by diverse methanogenic reservoir-associated microorganisms when susceptible hydrocarbons are present in crude oil.

## Materials and methods

### Sampling site description and sample collection

To prepare the inoculum for this study, produced water was obtained from five production wells (PW; 4-PW, 7-PW, 18-PW, 32-PW, 33-PW; Figure S1) in the Medicine Hat Glauconitic C (MHGC) field (Voordouw et al., 2009). Although this oilfield has been used as a study site for nitrate treatment of souring for 10 years (Suri et al., 2017), previous investigations of this oilfield have reported chemical and functional evidence of nitrate-free “zones” harboring active methanogenic archaea (Agrawal et al., 2012), from which all PW samples were collected for this study. Berdugo-Clavijo and Gieg (2014) also demonstrated that methanogenic crude oil biodegradation could be established from these produced waters. No detectable amounts of nitrate or sulfate, and only low concentrations of sulfide (0.05 mM), nitrite (0.01 mM), and ammonium (0.39 mM) were measured in the produced water samples (methods described in Voordouw et al., 2009). Therefore, we determined that the overall risk of nitrate/nitrite inhibition of methanogenesis was low and that PW samples could be used to study methanogenic crude oil biodegradation.

Samples were collected on May 20th, 2015 in 1-L Nalgene bottles filled to the brim to minimize oxygen ingress during transportation. Upon arrival in the lab, samples were stored in an anaerobic chamber (10% CO_2_/90% N_2_) at room temperature. Most samples contained 5–10% heavy oil (v/v), which was separated from the produced water by centrifugation (25,000 × *g*_av_ for 20 min). Recovered oil samples were combined and stored anaerobically at 4°C prior to use. The crude oil was not sterilized before use, but this appeared to have no effect on control (sterile) incubations established in this study as no methane was produced from sterile controls.

### Establishment of light and heavy oil-degrading produced water cultures

Oil-free produced water from all five PW samples was combined in equal ratios and 500 mL aliquots were dispensed into five sterile, custom-made glass vessels. Each bottle was outfitted with two Balch tube ports; one near the neck of a 1-L Schott flask and the second near its base (in the aqueous phase); the neck of the bottle was also sealed in glass to create an air-tight container (Figure S2). This design allowed for routine sampling of the headspace and culture fluids, respectively, without disturbing the oil-water transition zone. Cultures were amended with either a light oil (°API = 32) or MHGC heavy oil (°API = 16) in excess (20 mL) before sealing microcosms with butyl rubber stoppers and aluminum crimps. The intent of our experiment was not to completely biodegrade all susceptible crude oil components, but rather to observe the microorganisms and processes responding to amendment with either oil source. Cultures were incubated at MHGC reservoir temperatures (30°C) under dark and static conditions for 17 months. Note here that no supplemental growth medium, reducing agents or other culturing agents were added to cultures so as best to simulate the minimal nutrient availability of this particular reservoir. Due to the limited volumes of PW fluids collected during the sampling trip, only one live replicate was prepared per oil type so as to ensure that adequate sterile (autoclaved) replicates (one for each oil type) and an oil-free control (incubated for 21 months) could also be established.

### Chemical analyses

During the incubation period, microcosm headspaces were routinely monitored for methane production by gas chromatography (GC; Fowler et al., 2012). Additionally, cultures were subsampled in duplicate (2 × 25 mL) at designated time points (after 1, 2, 4, 8, 12, and 17 months) for chemical (metabolites) and DNA analyses. Following a centrifugation step (30,000 × *g*_av_), supernatants were acidified with 6 M HCl (pH < 2) and extracted and analyzed for putative hydrocarbon metabolites as silylated compounds by gas chromatography-mass spectrometry (GC-MS) following the procedure outlined by Berdugo-Clavijo and Gieg (2014). Putative hydrocarbon metabolites from silylated organic extracts were positively identified using MSD ChemStation software (version E.02.02.1431; Agilent Technologies) and by matching GC retention times and MS profiles to authentic standards purchased from Sigma Aldrich (97 – ≥99.5% purity) or that were chemically synthesized. Alkyl-substituted benzylsuccinic acids were made using the reflux reaction procedure described by Bickford et al. (1948), while an authentic standard of *n*-octylsuccinic acid was prepared by base hydrolysis (Kropp et al., 2000). Calibration curves of representative TMS-derivatized standards were used to quantify detectable (≥10 nM) hydrocarbon metabolites of interest (Berdugo-Clavijo and Gieg, 2014).

End point crude oil subsamples (1 mL) were collected from oil-amended cultures and sterile controls for hydrocarbon analysis. Though quantifying the extent of crude oil biodegradation was not a goal of our experiment, we were still interested in screening samples for any measurable hydrocarbon losses. Crude oil samples were diluted 1:10 in dichloromethane and analyzed in triplicate by GC-MS as described by Berdugo-Clavijo and Gieg (2014). Hydrocarbon loss was determined as a function of alkane or aromatic hydrocarbon to pristane or phenanthrene peak area ratios, respectively, as these components naturally present in both crude oils were deemed to be recalcitrant to degradation.

### Hydrocarbon activation gene analysis

Pelleted cells from subsampled culture fluids were extracted for genomic DNA using the FastDNA SPIN Kit for Soils (MP Biomedicals) and normalized to a concentration of 0.5 ng/μL. Each DNA extract was then probed for the presence of anaerobic hydrocarbon activation genes using a series of established primer sets and thermocycling conditions (Table 1). All PCR reactions (25 μL) were prepared with 12.5 μL 2x Master Mix (Fermentas; Thermo Fisher Scientific), 0.5 μL of each forward primer and corresponding reverse primer (10 μM), and 1 μL of template DNA. Purified amplicons of expected size were cloned using a commercial kit (TOPO TA; Thermo Fisher Scientific) and sent to Eurofins Genomics (Eurofins MWG Operon LLC, Huntsville, AL, USA) for Sanger sequencing. Trimmed consensus sequences of correct identity were queried against the NCBI non-redundant nucleotide database using BLASTn to identify homology to known sequences, and used to build bootstrapped maximum likelihood trees (500 replicates) in MEGA7 (Kumar et al., 2016). Retrieved hydrocarbon activation gene sequences were deposited in GenBank and are available under the accession numbers MG460804–MG460820.

**Table 1:** Amplification results for primer sets screened for targeted functional gene analysis of oil-amended produced water cultures.

**Primer name**		**Target gene**	**Primer sequence (5'−3')**	**Expected amplicon bp**	**Light oil**	**Heavy oil**	**References**
7772f 8546r		*bssA* s.l.	GACATGACCGACGCSATYCT TCGTCGTCRTTGCCCCAYTT	774	+	+	Winderl et al., 2007
Primer set 1	BssA327f BssA2004r	*bssA*	CGAATTCATCNTCGGCTACC GTCGTCRTTGCCCCAYTTNGG	1667	–	–	Washer and Edwards, 2007
Primer set 2	MBssA1516f BssA2524r	*bssA*	AGACCCAGAAGACCAGGTC ATGATSGTGTTYTGSCCRTAGGT	1008	–	–	
Primer set 3	BssA327f MBssA2446r	*bssA*	CGAATTCATCNTCGGCTACC ATGCTTTTCAGGCTCCCTCT	2119	–	–	
Primer set 5	BssA1985f BssA2524r	*bssA*	CNAARTGGGGCAAYGACGA ATGATSGTGTTYTGSCCRTAGGT	539	+	+	
Primer set 1	1294/1321f 1933/1981r	*assA, bssA*	TTTGAGTGCATCCGCCAYGGICT TCGTCRTTGCCCCATTTIGGIGC	*assA*: 661 *bssA*: 682	+	+	Callaghan et al., 2010
Primer set 2	1213f 1987r	*bssA*	GACATGACCGAYGCCATYCT TCRTCGTCRTTGCCCCAYTT	793	+	+	
Primer set 3	1294f (a) 1936r	*assA*	TTSGARTGCATCCGNCACGGN TCRTCATTNCCCCAYTTNGG	661	–	–	
Primer set 4	1294f (a) 2457r	*assA*	TTSGARTGCATCCGNCACGGN TTGTCCTGNGTYTTGCGG	1180	+	–	
Primer set 5	1294f (b) 1936r	*assA*	TTYGAGTGYATNCGCCASGGC TCRTCATTNCCCCAYTTNGG	661	–	–	
Primer set 6	1294f (b) 2457r	*assA*	TTYGAGTGYATNCGCCASGG TTGTCCTGNGTYTTGCGG	1180	+	–	
Primer set 7	1432f 1936r	*assA*	CCNACCACNAAGCAYGG TCRTCATTNCCCCAYTTNGG	523	–	+	
Primer set 8	1432f 2457r	*assA*	CCNACCACNAAGCAYGG TTGTCCTGNGTYTTGCGG	1042	–	–	
Primer set 9	1432f 1933/1981r	*assA, bssA*	CCNACCACNAAGCAYGG TCGTCRTTGCCCCATTTIGGIGC	523	–	–	
FAE-B	7768f 8543r	*bssA* s.l., *nmsA*	CAAYGATTTAACCRACGCCAT TCGTCRTTGCCCCAYTTNGG	775	+	+	von Netzer et al., 2013
FAE-N	7363f 7374f 8543r	*nmsA* s.str.	TCGCCGAGAATTTCGAYTTG TTCGAYTTGACGGACAGCGT TCGTCRTTGCCCCAYTTNGG	1180 1169	–	–	von Netzer et al., 2013
FAE-Kf	7757f-1 7757f-2 7766f 8543r	*assA*	TCGGACGCGTGCAACGATCTGA TCGGACGCGTGCAACGCCCTGA TGTAACGGCATGACCATTCT TCGTCRTTGCCCCAYTTNGG	786 786 777	–	–	von Netzer et al., 2013
assA2	1359–1376f 1785–1802r	*assA*	YATGWACTGGCACGGMCA GCRTTTTCMACCCAKGTA	426	–	+	Aitken et al., 2013
assA3	1394–1409f 1843–1860r	*assA*	CCGCACCTGGGTKCAYCA GKCCATSGTGTAYTTCTT	440	–	+	
Ncr2_for_ Ncr2_rev_		*Ncr*	TGGACAAAYAAAMGYACVGAT GATTCCGGCTTTTTTCCAAVT	320	–	–	Morris et al., 2014

*Sequence positions indicated for primers refer to the nucleotide position of the following references; Thauera aromatica K127 bss operon (Winderl et al., 2007; von Netzer et al., 2013), Azoarcus sp. strain T bssA (Washer and Edwards, 2007), and Desulfatibacillum alkenivorans AK-01 (Callaghan et al., 2010; Aitken et al., 2013). ncr primers (Morris et al., 2014) were designed from 2-naphthoyl-CoA reductase sequences retrieved from PAH-degrading strains N47 and NaphS2 (Eberlein et al., 2013; Boll et al., 2014). A (+) designates positive amplification using the specified primer, (–) for no amplification*.

### Quantification of fumarate addition genes

To capture and quantify as many fumarate addition genes (in the produced water cultures) as possible in a single assay, non-degenerate primers MHGC_bssAf (GACGACGGCTGCATGGA) and MHGC_bssAr (GCCTTCCCAGTTGGCGTA) targeting *bssA* were designed from aligned gene sequences retrieved from this study. Primer specificity was verified in Primer-BLAST (Ye et al., 2012), and PCR/qPCR products obtained using the non-degenerate primers (~708 bp) were cloned and sequenced to confirm that they targeted the correct gene. *bssA* gene abundance in technical duplicate DNA samples was determined in relation to calibration standards from a 10-fold dilution series (10^9^-10^1^ gene copies per μL) of PCR amplified *bssA* clones obtained in this study. qPCR reactions comprised SsoFast Evagreen Supermix (5 μL), PCR primers (1 μL of 10 pmoles/μL each), RNAse-free water (3 μL), and DNA template (1 μL), and were carried out using a BioRad CFX96 thermocycler as followed; an initial denaturation step (5 min at 94°C), up to 40 cycles of 1 min at 94°C and 1 min at 59.5 °C, and melt curve analysis (65–95°C with an increase of 0.5°C every 5 s). The efficiency in qPCR reactions of primer set MHGC *bssA* ranged between 95 and 105%, with *R*^2^-values for calibration curves >0.99. Reactions yielded a single amplification product, and log gene-abundance values for samples all fell within the linear range of the standard calibration. Gene abundances were also queried for the oil-free and sterile control DNA extracts. A similar procedure was attempted to design qPCR primers for *assA*, but they failed to amplify the target gene above threshold (quantifiable) levels; only in positive control tests was the expected quantification observed. We experienced the same problem when using established primer sets assA2Fq/assA2Rq described by Aitken et al. (2013).

### Microbial community analysis

Amplification and Illumina sequencing of extracted DNA was carried out by a two-step method targeting the V6-V8 regions of the 16S rRNA gene using universal primers 926Fi5 (TCGTCGGCAGCGTCAGATGTGTATAAGAGACAGAAACTYAAKGAATTGACGG) and 1392Ri7 (GTCTCGTGGGCTCGGAGATGTGTATAAGAGACAGACGGGCGGTGTGTRC). In the first round of PCR, reactions (25 μL) contained 12.5 μL 2x PCR Master Mix (Fermentas), 5 μL of each primer (1 μM) and 2.5 μL of gDNA template. PCR assays were performed using a three-step thermoprofile previously shown to obtain high amplicon yields from low concentrations of DNA (Klindworth et al., 2013): initial denaturation at 95°C for 5 min; 25 cycles of 95°C (40 s), 55.0°C (2 min), 72.0°C (1 min); final extension at 72°C for 7 min. In the second round of PCR, indices were added to amplicon ends using Nextera XT Index Kit primers (P5-S50X-OHAF and P7-N7XX-OHAF; Illumina). Reaction volumes were increased to 50 μL and contained 25 μL 2x PCR Master Mix, 5 μL of each primer (1 μM) and 10 μL of purified PCR I amplicon. Reaction conditions were modified from round I PCR: 95°C 3 min; 8 cycles of 95°C (30 s), 55.0°C (30 s), 72.0°C (30 s); 72°C 5 min. Amplicons of expected length were confirmed on a 1% agarose gel, purified using the Agencourt AMPure XP magnetic bead system (Beckman Coulter), and sequenced using the 300PE (paired-end) MiSeq protocol at the Department of Biological Sciences, University of Calgary.

Read assembly and 16S rRNA gene sequencing analysis were performed in QIIME (version 1.9.1; Caporaso et al., 2010). Prior to assembly, reads were visually inspected in DADA2 (Callahan et al., 2016) and trimmed of low-quality ends. Reads were merged with a 50 bp overlap with ≤10% allowed mismatches, and subjected to additional quality control steps such as the removal of chimeras, ambiguities and sequences with an average quality score of less than 20. The final reads were clustered into operational taxonomic units (OTUs) at a 97% species cutoff and classified against the SSU SILVA 119 database (Quast et al., 2013). The average read abundance of technical duplicates was used to profile microbial community compositions over time. Merged sequence reads were deposited to the National Center for Biotechnology Information Short Read Archive (SRA) under BioProject PRJNA417121 and accession numbers SAMN07977243–SAMN07977267.

## Results

### Methanogenic activity on light and heavy crude oil

**Figure 1:**
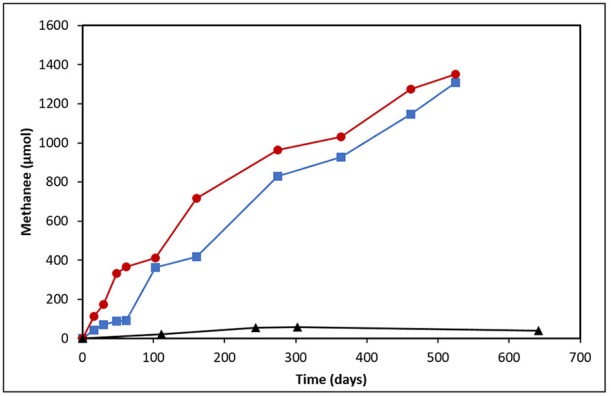
Methane production from produced water-derived incubations enriched on light (blue squares) and heavy (red circles) oil relative to an unamended control (black triangles).

Methane production was monitored following the establishment of two crude oil-amended incubations prepared from heavy oil reservoir produced water (Figure 1). The incubation amended with light oil produced near identical amounts of CH_4_ (1307 μmol) as the heavy oil-amended incubation (1352 μmol) in uniform rates across the 525-day incubation period (0.14 μmol CH_4_/day/g of light oil vs. 0.13 μmol CH_4_/day/g of heavy oil). The only deviation in CH_4_ production was in the first 60 days of incubation, where an apparent lag phase was observed in the light oil incubation.

**Figure 2:**
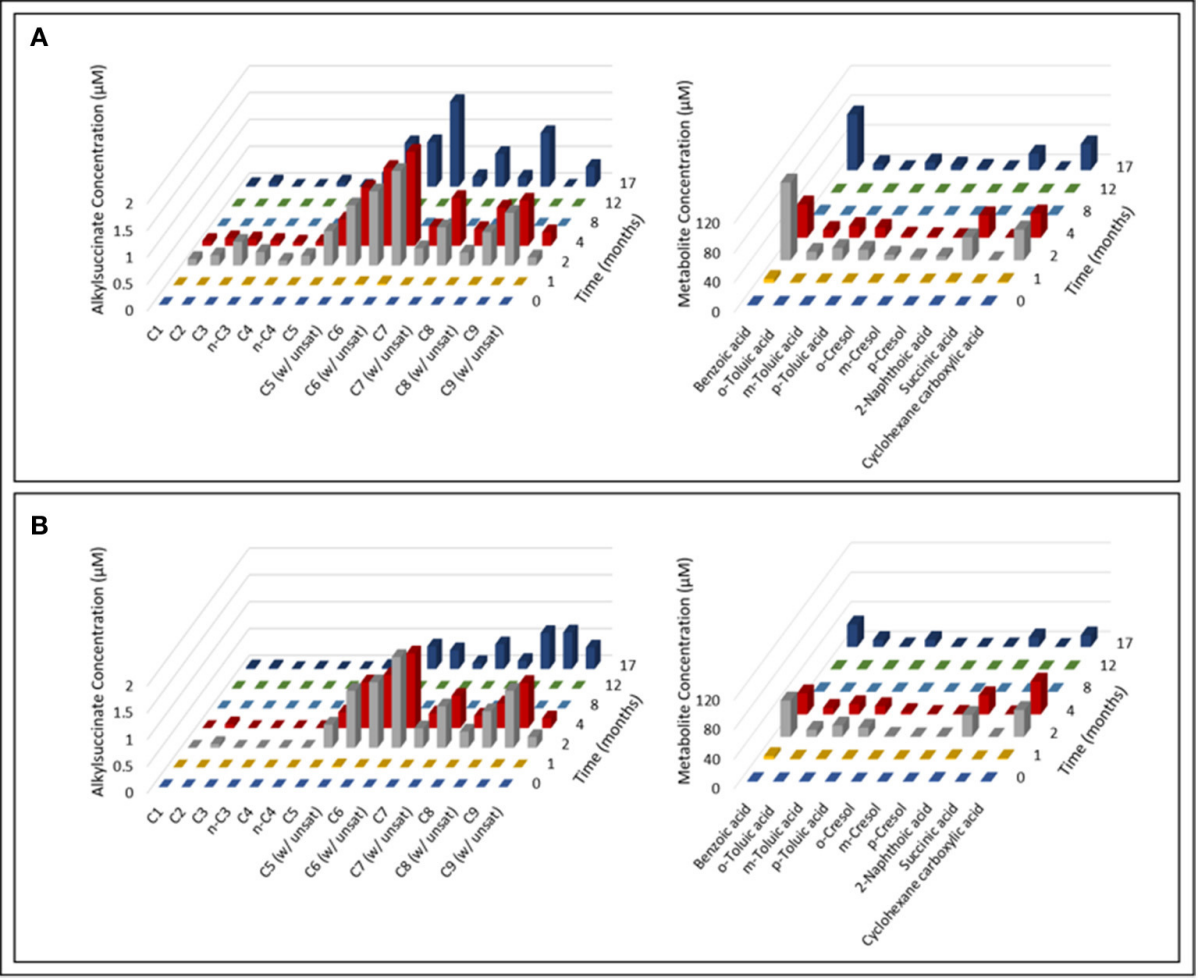
Time-resolved quantification of alkylsuccinic acids and aromatic hydrocarbon metabolites detected in the **(A)** light and **(B)** heavy oil-amended cultures. Characteristic ion fragments m/z 262 and (M – 15)^+^ were selected to probe and integrate TMS-derivatized alkylsuccinates and organic components, respectively. Identification and quantification of metabolites was performed using calibration curves prepared from authentic standards.

**Figure 3:**
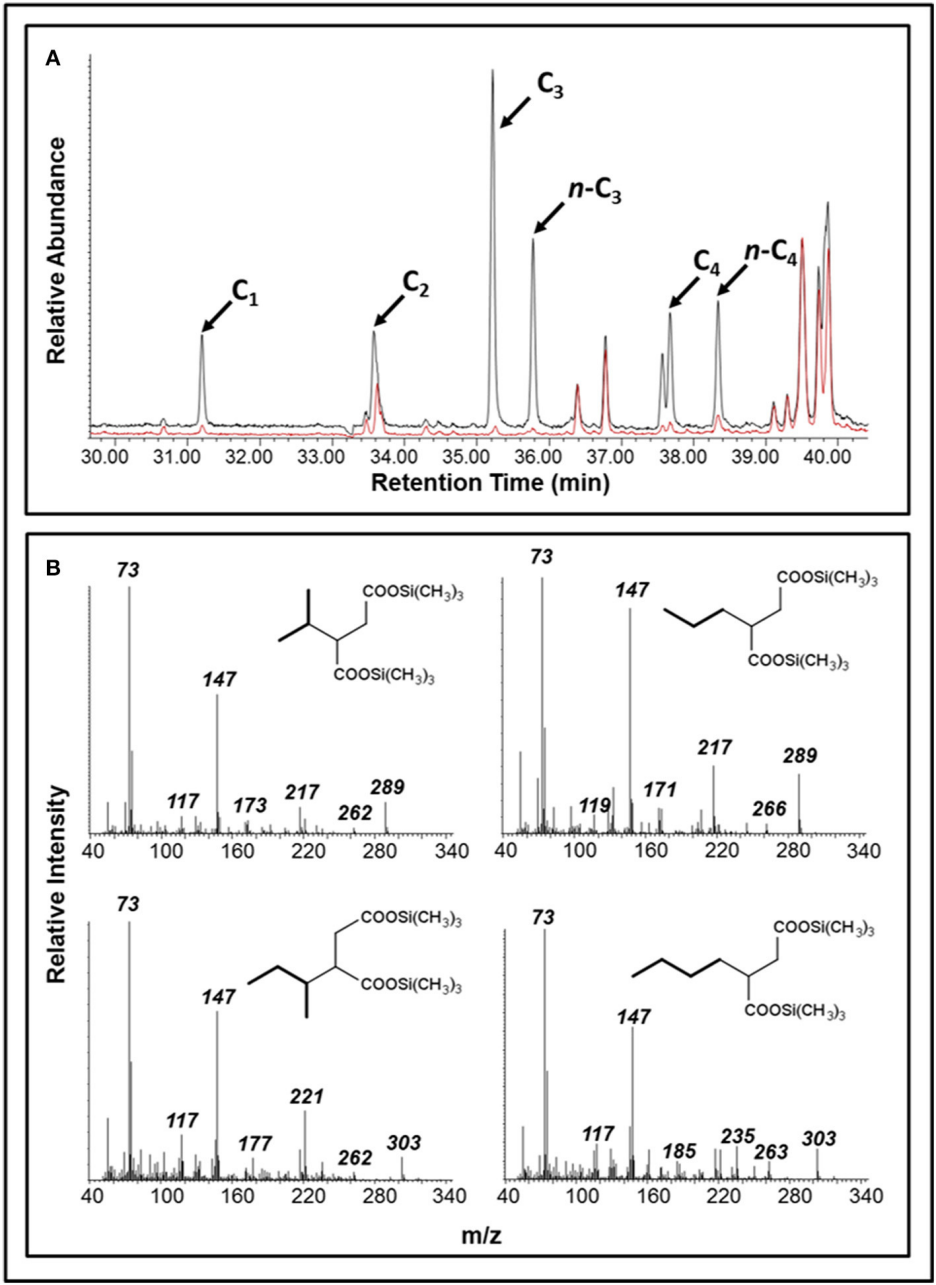
Detection of putative alkylsuccinates in oil-amended produced water cultures. **(A)** A portion of a GC total ion chromatogram showing larger peaks diagnostic of C_1_-C_4_ alkylsuccinates in the light oil culture (black) than in the heavy oil culture (red); peaks were not detected in the unamended control (not shown). **(B)** Mass spectral profiles indicative of propane and butane fumarate addition products at both the primary and secondary carbon atoms (shown as trimethylsilyl derivatives).

Over the course of the 17-month incubation period, we identified and quantified various compounds in both oil-amended cultures that were not present in the oil-free and sterile controls (Figure 2; Table S1). The average concentration of hydrocarbon metabolites in the oil-amended cultures peaked between 2 and 4 months of incubation, and included compounds with mass spectral profiles indicative of C_1_-C_9_ alkane fumarate addition (alkylsuccinates) and aromatic compound biodegradation (e.g., benzoate, toluates, 2-methylnaphthoate, cyclohexane carboxylate). No fumarate addition products for aromatic hydrocarbons could be detected in either culture at any time point. Generally, the concentration of aromatic acids detected in the light oil-amended culture was greater than in the heavy oil-amended culture (Figure 2). Trace amounts of C_1_-C_4_ alkylsuccinates (0.07–0.44 μM) were also predominantly detected in the light oil culture (Figures 2, 3). Interestingly, we identified a total of four peaks in the light oil-amended culture with MS fragment ions corresponding to propane or butane fumarate addition products (two each), suggesting that hydrocarbon activation was occurring at both the primary and secondary carbon atom (Figure 3; Kniemeyer et al., 2007). The mass spectral pattern of the putative *n*-propylsuccinate aligns with a previously published reference standard (Savage et al., 2010), but does require verification with an authentic standard in our laboratory. At T_8_ and T_12_, the concentration of most alkylsuccinates was below detectable limits (≤10 nM), and cumulative aromatic/other hydrocarbon metabolites also decreased to 2.6–7.6 μM; up to 52 times less than concentrations observed at T_2_ and T_4_ (Figure 2). Select metabolites were again detected by the end of the incubation period.

Triplicate oil samples from each oil-amended microcosm were recovered and assessed by GC-MS analysis after 17 months of incubation. Measurable losses of C_7_-C_8_ *n*-alkanes (47–79%), cyclohexane (22–71%), and some alkyl-substituted aromatic hydrocarbons were observed in oil-amended cultures as compared to sterile controls (Figure S3). The loss of these hydrocarbons corresponded well with hydrocarbon metabolites detected in the organic extracts (Figure 2). Other hydrocarbons detected in the incubations did not decrease in abundance relative to their corresponding sterile control (Figure S3), which we attribute to amending microcosms with an excess of oil.

### Detection of fumarate addition genes

Twenty established primer sets targeting anaerobic hydrocarbon activation genes were used to probe genomic DNA from the light- and heavy oil-amended cultures. Of these, 11 sets of primers could amplify *assA* or *bssA* gene fragments present in one or both cultures in at least one time point sample (Table 1), with the most amplification observed using primer sets 7772f/8546r (Winderl et al., 2007) and FAE-B (von Netzer et al., 2013). Purified and cloned amplicons of target genes were sequenced to determine the taxonomic affiliation of each PCR product and to assess the diversity of putative hydrocarbon degraders in each culture. In all, 3 unique *assA* and 14 *bssA* gene fragments were retrieved across both oil-degrading microcosms; PCR products for polycyclic aromatic hydrocarbon (PAH) activation genes (*nms* encoding for naphthylmethylsuccinate synthase and *ncr* encoding for naphthyl-coenzyme A reductase) were not obtained at any time point.

**Figure 4:**
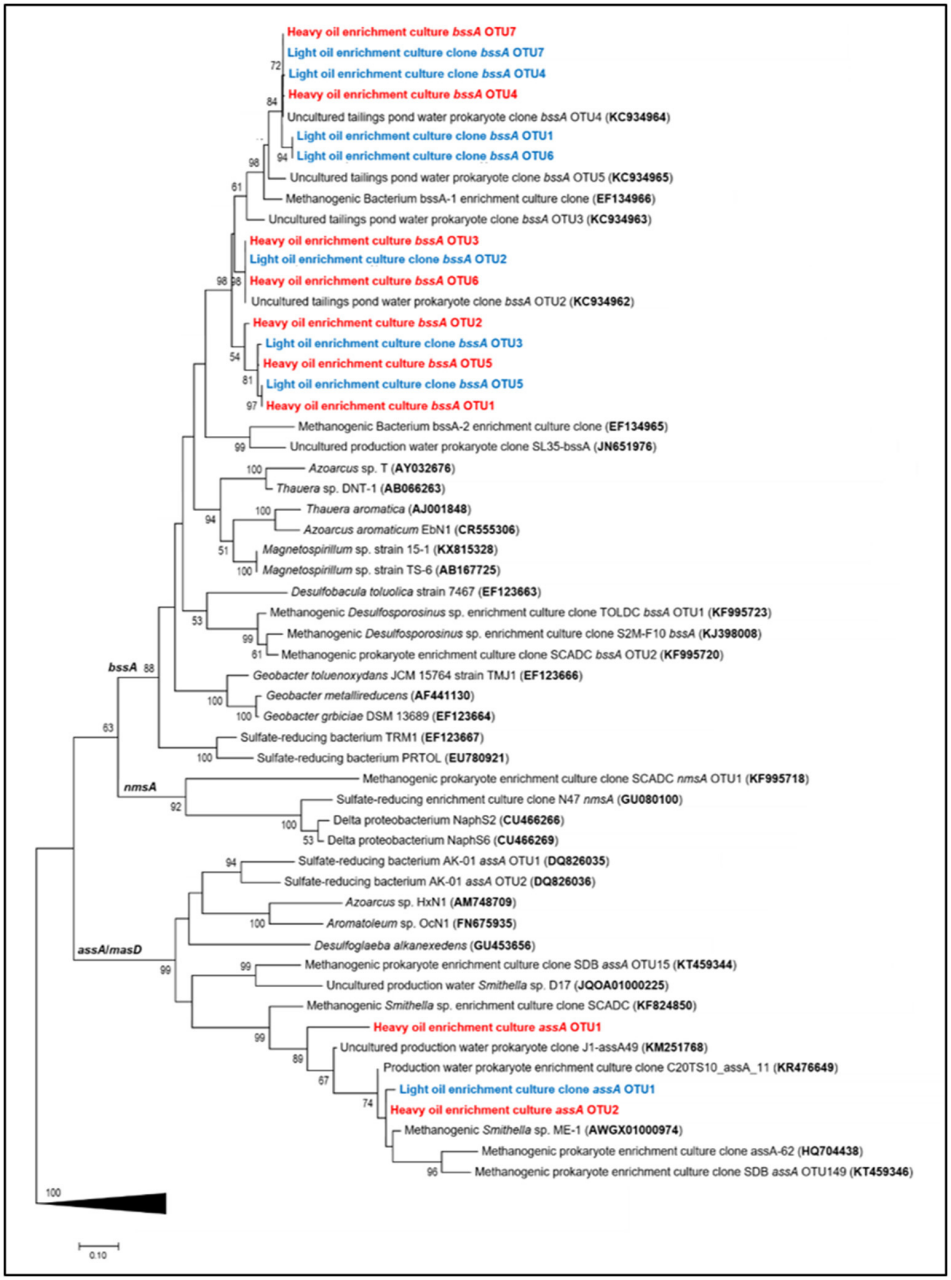
Maximum likelihood tree showing the affiliation of recovered *assA* and *bssA* gene fragments (this study, light oil in blue and heavy oil in red) with previously published reference strains, enrichment cultures, and environmental samples. Evolutionary analyses of aligned nucleotide sequences were conducted in MEGA7 (Kumar et al., 2016); the consensus tree was constructed using the Tamura–Nei model (Tamura and Masatoshi, 1993) at all nucleotide positions (for a total of 481 positions in the final dataset) and performing 500 bootstrap replicates (values below 50% are not shown). Pyruvate formate lyase (*pfl*) sequences were used as an outgroup (collapsed in figure).

Maximum likelihood trees of the recovered fumarate addition gene fragments revealed that all *assA* gene sequences clustered within a *Smithella* subclade predominantly enriched from alkane and/or crude oil substrates, whereas *bssA* sequences were distributed within a largely uncharacterized clade (Figure 4). The *bssA* clade was phylogenetically distinct from published fumarate addition gene sequences belonging to cultured aromatic hydrocarbon degraders (<77% sequence similarity), thus we assessed their taxonomic affiliations to previously characterized enrichment cultures and environmental strains. We found that the *bssA* gene fragments retrieved from the oil-degrading cultures shared 90–91% sequence similarity to a presumed *Desulfotomaculum* sp. previously recovered from a methanogenic toluene-degrading enrichment culture (Bacterium bssA-1; Edwards and Grbić-Galić, 1994; Washer and Edwards, 2007). Our *bssA* gene fragments also clustered closely (92 – 99% sequence similarity) to several uncultured prokaryotic clones recovered from the Mildred Lake Settling Basin, a tailings pond located in Northern Alberta, Canada, from which materials were used to establish a series of hydrocarbon-degrading enrichment cultures (e.g., Siddique et al., 2006, 2007, 2011; Tan et al., 2013, 2015a).

### Time-resolved quantification of fumarate addition genes

To gain a better understanding of *assA* and *bssA* activity in the oil-degrading cultures, fumarate addition gene abundances were estimated over time by qPCR analysis. Though we were able to detect *assA* genes in the previously mentioned PCR assays, we were unable to quantify them using published qPCR primers (Aitken et al., 2013) and experimental primers; only positive control tests yielded a quantifiable amplicon product. Consequently, changes in *assA* gene abundance over time could not be evaluated. It is not known whether the abundance of *assA* is indeed below detectable limits (<10^1^ gene copies/μL), or if the primers selected failed to capture all phylotypes present in the DNA extracts.

**Figure 5:**
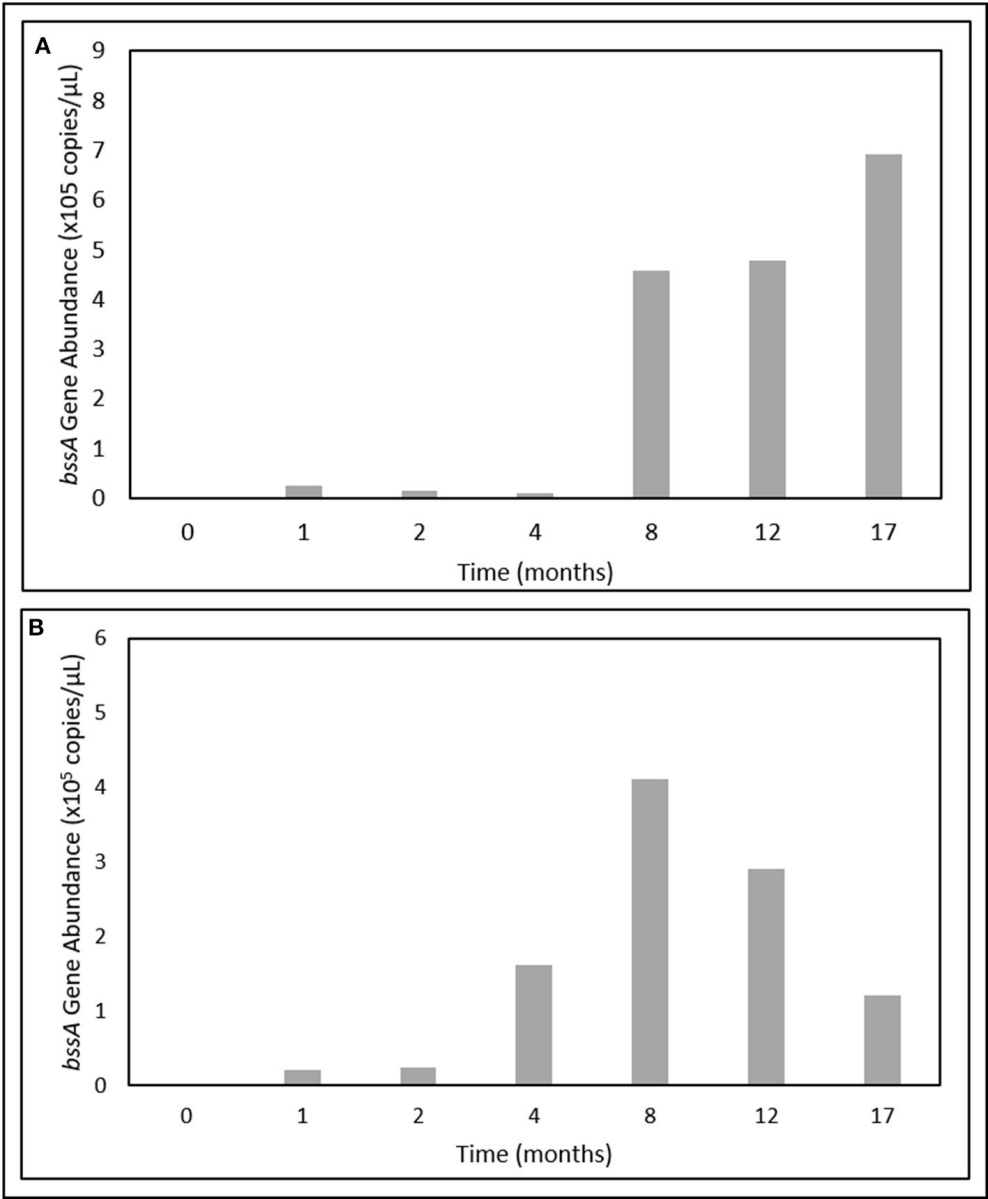
Change in *bssA* gene abundances over time in the **(A)** light and **(B)** heavy oil-amended produced water cultures.

In contrast, *bssA* gene fragments were successfully quantified over the 17-month incubation period. Gene abundances were below detectable limits at T_0_, but became enriched after only 1 month of incubation (Figure 5). In the light oil culture, *bssA* gene abundances increased > 25-fold between T_4_ and T_8_, and continued to increase up to a maximum of 6.92 × 10^5^ copies/μL (Figure 5A). While *bssA* gene abundances in the heavy oil culture also increased during the first 8 months of incubation, values decreased by 70% over the remaining incubation period (Figure 5B).

### Microbial community dynamics in methanogenic cultures

**Figure 6:**
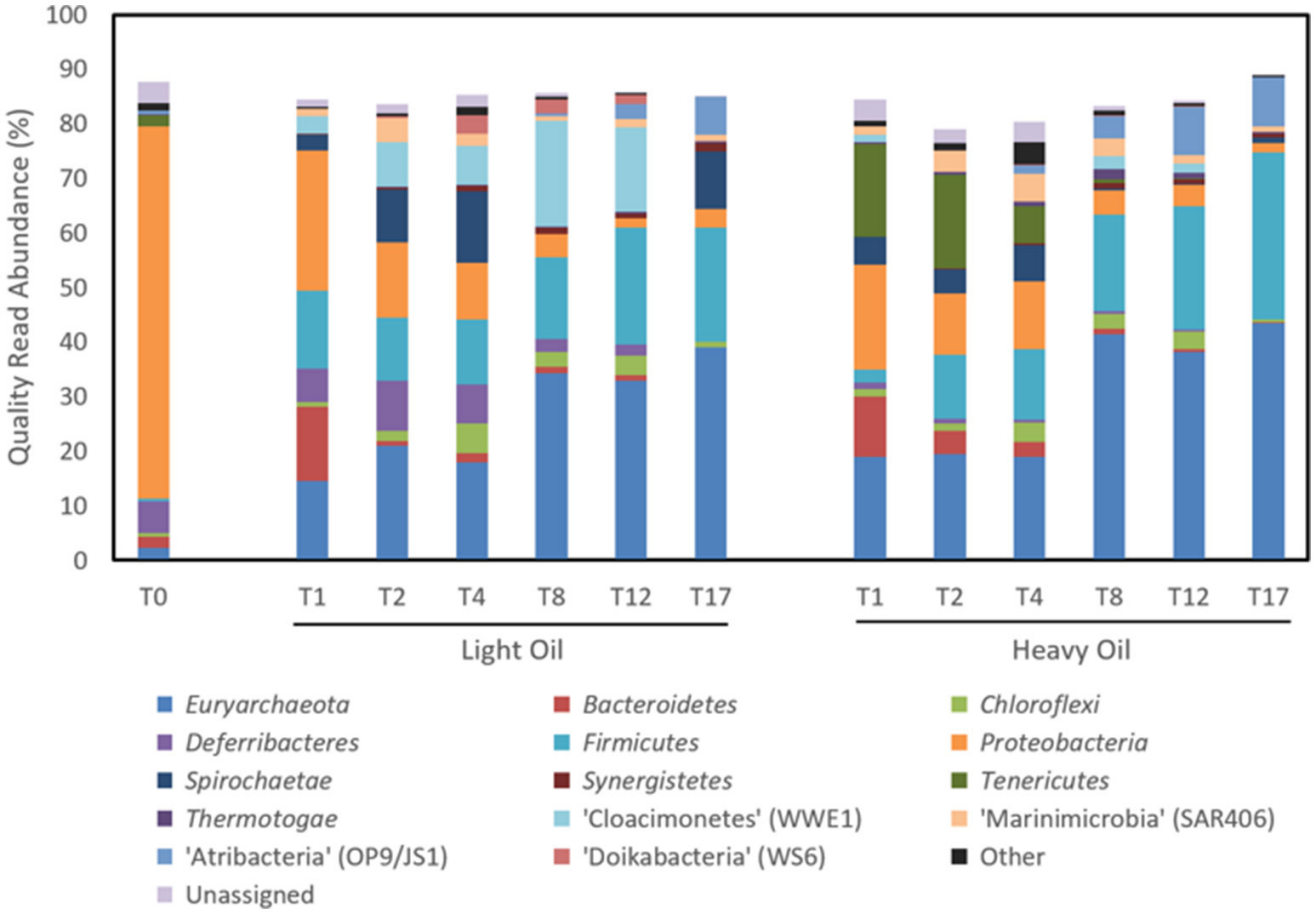
Microbial community composition of methanogenic crude oil-degrading produced water cultures over time at the phylum level based on 16S rRNA gene sequencing.

Time-resolved sequencing of 16S rRNA gene fragments was carried out to compare microbial community dynamics in response to amendment with light and heavy oil (Figure 6, Table 2). Both the light and heavy oil cultures saw substantial shifts in microbial community composition during the 17-month incubation under simulated reservoir conditions: many of these shifts were similar across each culture, but distinctions were also evident. The most apparent increase in abundance over time was seen within the *Firmicutes* and *Euryarchaeota* phyla, comprising 60 and 74% of quality sequence reads in both oil-amended cultures by T_17_, respectively (Figure 6). This represented a 20- to 25-fold increase in abundance from reported T_0_ values, which collectively had comprised less than 3% of total reads (Figure 6). Members of the *Firmicutes* were dominated by the enrichment of a single OTU affiliated with *Desulfotomaculum* after T_4_, making up to 30.5% of reads by the end of the 17-month incubation period (Table 2). This OTU shared >97% sequence similarity to an uncultured *Peptococcaceae* bacterium clone recovered from the Mildred Lake Settling Basin (accession no. EU22655; Siddique et al., 2012); a bacterium thought to participate in methanogenic hydrocarbon biodegradation. This trend also closely mirrored the increase in *bssA* gene abundances over time, particularly in the light oil-amended culture (Figure 5). Thus, it is plausible that (at least some of) the *bssA* gene sequences retrieved from our cultures belong to *Desulfotomaculum* and/or the *Peptococcaceae* family. Other members of the *Firmicutes* were also enriched (though to a lesser extent) during the first 4 months of incubation, including *Dethiosulfatibacter* (up to 8.8% of light oil reads) and *Moorella* (up to 10.0% of heavy oil reads), but their relative abundance decreased to <0.1% immediately afterwards (Table 2). Methanogenic *Euryarchaeota* became enriched after the first month of incubation and proliferated up to 39.1–43.3% of total reads by T_17_ (Figure 6), including hydrogenotrophic (*Methanocalculus, Methanoculleus*, and *Methanolinea*) and acetotrophic (*Methanosaeta*) representatives (Table 2). Though the ratios of methanogens are similar between both cultures, a greater proportion of reads belonging to *Methanocalculus* were enriched over time in light oil-amended samples, whereas *Methanolinea* was up to three times as prevalent in the presence of heavy oil at T_17_. Other putative hydrocarbon fermenters and/or hydrocarbon degradation-associated bacteria were detected during the incubation period, including microorganisms affiliated with the *Peptococcaecae, Anaerolineaceae*, or *Syntrophaceae* families (averaging 2.5% of reads; Table 2). We also detected an OTU belonging to *Smithella* (up to 6.1% of reads), but unlike *Desulfotomaculum*, its read abundance declined after 4 months of incubation. Several of the T_0_ reservoir-associated OTUs, consisting primarily of *Proteobacteria*, were reduced to <0.5% of reads after just the first month of incubation (includes members of the *Alpha*-, *Beta*-, and *Gammaproteobacteria*), while others saw a more gradual decrease in abundance over time (e.g., *Deltaproteobacteria, Deferribacteres*; Figure 6; Table 2).

**Table 2:** Distribution of the 25 most abundant classified taxa (%) across both methanogenic crude oil-degrading produced water cultures over 17 months of incubation, as determined by Illumina sequencing of the 16S rRNA gene.

**Taxon**		**Light oil**						**Heavy oil**					
	**T_0_**	**T_1_**	**T_2_**	**T_4_**	**T_8_**	**T_12_**	**T_17_**	**T_1_**	**T_2_**	**T_4_**	**T_8_**	**T_12_**	**T_17_**
**METHANOGENS**													
*Methanocalculus*	0.2	8.6	13.1	5.3	19.4	13.6	18.1	2.0	1.4	4.3	16.8	10.9	9.6
*Methanosaeta*	0.0	0.0	0.5	8.8	4.4	9.9	9.4	2.3	4.7	4.9	7.7	9.9	12.5
*Methanoculleus*	1.8	2.3	2.3	1.0	2.2	3.8	7.8	9.0	7.7	3.2	5.0	5.7	8.3
Uncultured *Methanoregulaceae*	0.0	0.1	0.1	0.3	7.2	4.2	0.0	0.5	0.0	1.5	5.9	6.2	1.3
*Methanofollis*	0.0	2.8	3.9	1.8	0.4	0.3	0.3	2.8	3.1	2.7	1.7	1.6	1.2
*Methanolinea*	0.0	0.0	0.0	0.0	0.0	0.0	3.2	0.0	0.0	1.1	2.4	2.2	9.0
**HYDROCARBON DEGRADATION-ASSOCIATED BACTERIA**													
*Desulfotomaculum*	0.4	0.0	0.0	0.0	14.5	21.2	21.0	0.0	0.0	0.1	17.2	22.4	30.5
Uncultured *Peptococcaceae*	0.0	0.0	0.1	0.1	0.0	0.0	0.0	0.0	0.2	2.7	0.4	0.2	0.0
Uncultured *Anaerolineaceae*	0.2	0.9	1.9	5.3	2.5	3.5	0.9	0.9	1.1	2.9	2.6	3.1	0.6
*Smithella*	0.0	0.0	2.7	2.8	0.0	0.0	0.2	0.0	2.7	6.1	0.0	0.0	0.6
Uncultured *Syntrophaceae*	0.0	5.0	2.0	0.8	0.5	0.6	0.3	0.3	1.4	2.6	1.3	0.8	0.4
*Syntrophus*	0.4	1.3	0.1	0.2	0.4	0.4	0.00	1.3	0.3	1.0	1.8	1.4	0.1
**CELL MATERIAL/ORGANIC ACID SCAVENGERS**													
*Pelobacter*	3.3	15.9	6.6	3.9	0.1	0.1	0.0	12.0	4.0	0.8	0.3	0.2	0.0
Uncultured *Spirochaetes*	0.0	0.0	7.8	12.3	0.0	0.0	10.6	0.0	1.8	5.7	0.0	0.0	1.0
Uncultured *Bacteroidales*	0.5	12.3	0.5	1.0	0.9	0.5	0.0	8.6	0.0	0.0	0.7	0.4	0.0
*Dethiosulfatibacter*	0.0	8.8	8.1	7.2	0.5	0.3	0.0	0.0	0.0	0.1	0.0	0.0	0.0
Uncultured *Deferribacteraceae*	2.6	0.4	9.3	7.1	0.0	0.0	0.0	0.0	0.7	0.6	0.0	0.0	0.1
*Moorella*	0.0	0.0	0.0	0.0	0.0	0.0	0.0	0.0	10.1	8.7	0.0	0.0	0.1
*Desulfomicrobium*	3.6	1.8	1.4	0.9	0.1	0.0	0.0	3.0	1.7	1.5	0.2	0.1	0.0
**NRB-ASSOCIATED**													
Uncultured *Rhodocyclaceae*	13.8	0.1	0.0	0.0	0.0	0.0	0.0	0.0	0.0	0.0	0.0	0.0	0.0
*Flexistipes*	0.5	5.7	0.0	0.0	2.3	1.9	0.0	0.7	0.0	0.0	0.5	0.4	0.0
*Thauera*	10.4	0.1	0.0	0.0	0.0	0.0	0.0	0.1	0.0	0.0	0.0	0.4	0.0
**UNKNOWN**													
“Cloacimonetes” (WWE1)	0.0	3.2	0.0	0.0	19.4	15.4	0.0	1.4	0.0	0.0	2.5	1.6	0.0
“Atribacteria” (OP9/JS1)	0.6	0.0	0.0	0.0	0.4	2.8	7.0	0.0	0.0	1.4	4.1	8.8	9.1
“Marinimicrobia” (SAR406)	0.0	1.4	4.5	2.2	0.9	1.5	1.1	1.5	3.9	5.1	3.2	1.6	0.9
Total	38.3	70.7	64.9	61.0	76.1	80.0	79.9	46.4	44.8	57.0	74.3	77.9	85.3

*Taxa are sorted by their inferred community role. Inset heat map denotes time points with the highest relative abundance of each taxon across both cultures, whereby an increasing abundance of a given taxon is indicated by an increasingly darker shade of green*.

Other OTUs predominantly affiliated with known protein/or organic acid scavengers (e.g., *Pelobacter, Bacteroidales*) saw a rapid increase in abundance (up to 39.4% of reads) at T_1_, and immediately declining afterwards (Table 2). Interestingly, three candidate phyla were enriched at different times during the incubation period. Most notably, the proliferation of “Atribacteria” (formerly OP9/JS1) paralleled that of *Desulfotomaculum*, increasing by up to 7.0–9.1% over 17 months (Figure 6; Table 2). In contrast, the enrichment of “Cloacimonetes” (WWE1) peaked near T_8_, and “Marinimicrobia” (SAR406) remained between 1.1 and 5.1% abundance after T_0_ (Figure 6; Table 2).

## Discussion

Crude oil degradation in deep reservoir environments over geological time has been attributed to methanogenesis, a process that still requires a deeper understanding. Here we assessed the functional and microbial community response of an oilfield produced water consortium over time following exposure to light or heavy oil. Our cultures were found to behave similarly to amendment with either crude oil source, whereby fermentative bacteria (e.g., *Desulfotomaculum, Smithella*) were found to catalyze the activation of susceptible low molecular weight hydrocarbons (e.g., short-chain *n*-alkanes, cyclohexane and monoaromatic hydrocarbons) via addition to fumarate. This study shows that fumarate addition is a possible activation mechanism to catalyze crude oil biodegradation by reservoir-associated microorganisms, building on initial reports of methanogenic hydrocarbon degradation from the MHGC field (Agrawal et al., 2012; Berdugo-Clavijo and Gieg, 2014) and microbial communities enriched from other oilfield fluids (Jones et al., 2008; Gieg et al., 2010; Mbadinga et al., 2012; Zhou et al., 2012; Cheng et al., 2013; Tan et al., 2013).

The detection of enhanced methane production in each oil-amended culture relative to the oil-unamended control (Figure 1), in addition to the increase in abundance of methanogenic archaea over time (Figure 6), shows that the MHGC field continues to harbor methanogenic hydrocarbon-degrading microbial communities (Agrawal et al., 2012; Berdugo-Clavijo and Gieg, 2014) despite its history of nitrate treatment for souring (Voordouw et al., 2009; Suri et al., 2017). Though this does not rule out the possibly that methanogenesis has been (at least) partially impacted by nitrate/nitrite over time, nitrate treatment has not prevented methanogenic crude oil biodegradation from occurring in our culture experiments. We remark that experimental rates of methane production from crude oil were 30–60 times slower than in a previous study examining methanogenic hydrocarbon degradation using MHGC production water (Berdugo-Clavijo and Gieg, 2014). However, a key difference is that nutrients (including 1 mM phosphate) were added in the previous work, while in the present study no additional nutrients were added. Thus, lower nutrient availability may partially explain the slower rates of methane production. Volatile hydrocarbons present in light oils (e.g., *n-*C_5_–*n*-C_10_ alkanes, methylcyclohexane, benzene, toluene, and xylenes) are known to partially inhibit methanogenic hydrocarbon biodegradation (Sherry et al., 2014) and may have contributed to the 2-month lag in methane production seen in the light oil culture, but had no apparent long-term effect (Figure 1).

The detection of known anaerobic hydrocarbon metabolites (including fumarate addition products) offers convincing evidence that biodegradation processes are occurring in anoxic environments, and can provide clues as to the mechanism(s) responsible for their transformation (Gieg and Suflita, 2002; Gieg and Toth, 2017). Using a combined approach of metabolite analysis and targeted functional gene analysis in a time-dependent fashion, we present evidence that fumarate addition is a possible mechanism of hydrocarbon activation by MHGC-associated microbial community members, and that methanogenic degradation of low molecular weight *n*-alkanes (≤C_8_), monoaromatic hydrocarbons, and possibly cyclic alkanes was occurring simultaneously in each culture. Further, this study demonstrates the importance of using a combined, time-dependent approach of metabolite and biodegradation gene analysis in order to compensate for detection and specificity limitations in chemical and molecular assays. For example, conducting qPCR of *bssA* (detection limit of 10^1^ copies/μL) demonstrated an increase in abundance of this fumarate addition gene involved in alkylaromatic metabolism as methanogenesis progressed (Figures 1, 5), although the requisite benzylsuccinates could not be detected (<10 nM). Similarly, alkylsuccinates diagnostic of saturated alkane activation could be detected in the cultures and *assA* gene(s) could be detected in the cultures (Figures 3, 4), although a qPCR assay could not be devised to quantify the requisite *assA* gene above detection levels. Similar anomalies have been reported in other studies that aimed to demonstrated anaerobic hydrocarbon metabolism (Beller et al., 2008; Aitken et al., 2013; Johnson et al., 2015), underlining the importance of utilizing a multi-pronged approach in order to garner evidence for anoxic hydrocarbon-degrading metabolic processes in environmental samples.

An accumulation of hydrocarbon metabolites (e.g., alkylsuccinates, aromatic acids) was observed by the second month of incubation, and persisted for at least 2 months before decreasing to trace- or below-detectable amounts (Figure 2). We propose that the accumulation of metabolites was due to the microbial community adaption from predominantly denitrifiers (maintained by nitrate injection in the MHGC oil field, e.g., uncultured *Rhodocyclaceae* and *Thauera*; Agrawal et al., 2012) to predominantly methanogenic consortia (Figure 6; Table 2), requiring several months of incubation before active syntrophic hydrocarbon degradation finally proceeded. Research groups have typically assessed enrichment cultures for hydrocarbon metabolites well after methanogenic activity has already been established (e.g., Aitken et al., 2013; Tan et al., 2013; Berdugo-Clavijo and Gieg, 2014), when these intermediates are being or have been rapidly consumed. This approach can make it difficult to detect putative hydrocarbon metabolites using standard GC-MS approaches, as seen by Aitken et al. (2013), especially when extracting small culture volumes. Thus, it may be a better approach to look for metabolites early in a time-course experiment, when metabolites appear to be at their highest concentrations (Figure 2). It is not clear why some metabolites appear to re-accumulate over time, but this may be due to natural fluctuations in the community composition impacting the abundance and/or metabolic activity of fermentative (syntrophic) partners (e.g., *Smithella, Syntrophus*), which we found to be dynamic throughout the time course experiment (Table 2). The quantitative significance of such *Syntrophaceae*, particularly in methanogenic alkane-degrading communities, has been a topic of interest in recent years (e.g., Gray et al., 2011; Cheng et al., 2013; Embree et al., 2013; Wawrik et al., 2016). For example, Fowler et al. (2014) proposed a *Syntrophus* sp. to be a key secondary syntroph in the methanogenic toluene-degrading culture TOLDC, consuming the intermediate benzoic acid produced by a *Desulfosporosinus* sp., but at a slower rate. This might also explain why downstream metabolites of aromatic hydrocarbon degradation could be detected in each oil-amended culture, but not a fumarate addition product corresponding to *bssA* genes recovered from extracted DNA (Figures 2–4). Interestingly, we obtained putative mass spectral evidence that the short chain *n*-alkanes propane and butane present in the light oil culture underwent hemolytic C-H bond cleavage by addition to fumarate at the C_2_ or terminal carbon position (Figure 3). This has been reported in a few other instances by microbiota enriched or isolated (strain BuS5) from hydrocarbon seeps (Kniemeyer et al., 2007; Savage et al., 2010) or in production fluids (Bian et al., 2015; Gruner et al., 2017). Fumarate addition has also been reported to occur to a lesser extent at C_3_ for larger alkanes (Rabus et al., 2001), though it has been hypothesized that *n*-alkylsuccinates are formed accidentally during fumarate addition rather than as true intermediates (Rabus et al., 2001; Jarling et al., 2015).

Phylogenetic and functional gene evidence indicates that *Desulfotomaculum* is a key alkyl-substituted aromatic hydrocarbon degrader in each oil-amended culture (Figures 4–6; Table 2), a phylotype previously detected in MHGC produced water enriched on a light oil (°API = 37; Berdugo-Clavijo and Gieg, 2014) but at a lower abundance (3.3% of reads after 10 months of incubation). Members of the genus *Desulfotomaculum* can be metabolically versatile. They are commonly found in the subsurface biosphere through culture-based and molecular approaches, and have been found to participate in the degradation of alkanes (Kniemeyer et al., 2007; Cheng et al., 2013), aromatic hydrocarbons (Ficker et al., 1999; Morasch et al., 2004; Abu Laban et al., 2009; Berlendis et al., 2010; Selesi et al., 2010), and biphenyl (Selesi and Meckenstock, 2009). Though commonly known as sulfate-reducers, some species are capable of oxidizing various substrates (e.g., carbohydrates, organic acids and H_2_, among others) using other sulfur-containing compounds or metals as electron acceptors (reviewed by Aüllo et al., 2013). Other members of the *Desulfotomaculum* (cluster I) have lost the ability for sulfate respiration and instead grow syntrophically in concert with methanogens (Imachi et al., 2006). These phylotypes have been increasingly detected in petroleum reservoirs and in hydrocarbon-containing environments (Gieg et al., 2008; Tan et al., 2015b; Hu et al., 2016) where they presumably act as either primary or secondary syntrophs (Imachi et al., 2006; Kleinsteuber et al., 2012). Compared to published *Desulfotomaculum* cluster I sequences (Imachi et al., 2006), the *Desulfotomaculum* 16S rRNA and *bssA* genes recovered from the produced water cultures clustered within Ii (data not shown); a largely uncharacterized clade containing uncultured prokaryotic clones with the genetic potential to activate and subsequent degrade alkanes and monoaromatic hydrocarbons via fumarate addition (Tan et al., 2015b; Hu et al., 2016). We also observed an increase in *bssA* gene abundances coinciding with the increase in *Desulfotomaculum* reads over time (particularly in the light oil-amended culture; Figures 4, 5; Table 2). It is not currently known why the *bssA* gene abundance decreased in the heavy oil culture after 8–12 months of incubation, despite the continuous increase in *Desulfotomaculum* reads (Figure 6; Table 2), but may be due to PCR primer/sequencing biases not capturing all *Desulfotomaculum* phylotypes in the crude oil incubations. There may also be other organisms enriched after these time points that also catalyze aromatic hydrocarbon degradation, such as members of candidate divisions (discussed below), but whose functional genes were not captured using the marker gene assays screened in this study (Table 1).

From our microbial community sequencing results, we also hypothesize that the candidate phylum “Atribacteria” (formerly known as OP9/JS1) and other enriched candidate phyla play a chief role in methanogenic hydrocarbon metabolism based on their progressive enrichment or relative stability over time (Figure 6; Table 2). A recent study by Hu et al. (2016) discussed the role of candidate phyla in the biodegradation of crude oil, finding that “Atribacteria” dominated samples retrieved from oil reservoirs that exhibited the most extensive crude oil biodegradation (while candidate phyla in the other, less-biodegraded samples comprised less than 0.4% abundance in each sample). Sequence fragments orthologous to benzylsuccinate synthase (alpha, gamma subunits), (1-methyl)alkylsuccinate synthase (alpha subunit) and several glycyl radical enzyme activation proteins were identified from “Atribacteria” bins recovered from metagenomic sequencing of the Alaskan oil reservoir samples interrogated (Hu et al., 2016). Carr et al. (2015) also found “Atribacteria” in a methane-rich environment and suggested that it produced methanogenic substrates such as acetate and CO_2_. Interestingly, Hu et al. (2016) also prepared a draft genome for “Marinimicrobia” (SAR406) and identified an Fe-containing hydrogenase within its genetic material, thus the microorganism may produce hydrogen and participate in syntrophic interactions with methanogens. Members of the phylum “Cloacimonetes” (WWE1) were recently identified in fluids collected from coalbed methane wells (Kirk et al., 2015) and in anaerobic sludge digesters (Limam et al., 2014), also indicating a putative hydrocarbon degrading role for these phylotypes. Most convincingly, Cheng et al. (2013) identified members of the “Cloacimonetes” that participate in ^13^C-hexadecane biodegradation within a methanogenic oilfield enrichment consortium (along with *Syntrophaceae*).

Overall, the results from the present study indicate that fumarate addition is a possible mechanism of methanogenic hydrocarbon activation by oilfield-associated microbial communities and as such may represent a key metabolic pathway contributing to heavy oil formation in petroleum reservoirs. Further, time course-based functional gene analyses and microbial community sequencing identified *Desulfotomaculum* as a key aromatic hydrocarbon degrader under methanogenic conditions. Members of several candidate divisions may also play important roles in methanogenic hydrocarbon degradation, expanding on existing knowledge of the diversity of hydrocarbon-degrading consortia. To assess the putative metabolic functions of “Atribacteria” and other candidate phyla enriched in this study, the metagenome of the light oil-amended culture (from DNA extracted at T_12_) was recently sequenced using Illumina MiSeq technology; read assembly and analysis are intended future work. We intend to use this metagenomic data to help to uncover putative alkane/hydrocarbon degraders in these communities (e.g., *Smithella*), which have not yet been conclusively identified in this microbial community, and to help design primers that can capture a greater diversity of anaerobic hydrocarbon degradation genes.

## Author contributions

CT and LG conceived this research as part of a larger collaboration at the University of Calgary. CT established the oil-degrading produced water cultures and conducted all of the experimental procedures and analyses detailed in this study. CT and LG prepared the manuscript.

### Conflict of interest statement

The authors declare that the research was conducted in the absence of any commercial or financial relationships that could be construed as a potential conflict of interest.

## Footnotes

^1^https://www.bp.com/en/global/corporate/energy-economics/statistical-review-of-world-energy/oil/oil-reserves.html.
