# Supplementary material for: Time Course-Dependent Methanogenic Crude Oil Biodegradation: Dynamics of Fumarate Addition Metabolites, Biodegradative Genes, and Microbial Community Composition
Source: Front Microbiol. 2018 Jan 4;8:2610. doi: 10.3389/fmicb.2017.02610 (PMC5758579; doi:10.3389/fmicb.2017.02610)
Supplement: Supplementary file 1 [file Presentation1.pdf]

SUPPLEMENTARY MATERIALS

Time course-dependent methanogenic crude oil biodegradation: dynamics of fumarate addition metabolites, biodegradative genes, and microbial community composition

Courtney R. A. Toth[1], and Lisa M. Gieg[1]

[1]Petroleum Microbiology Research Group, Department of Biological Sciences, University of Calgary, Calgary, AB, Canada

3 Supplementary Figures

1 Supplementary Table

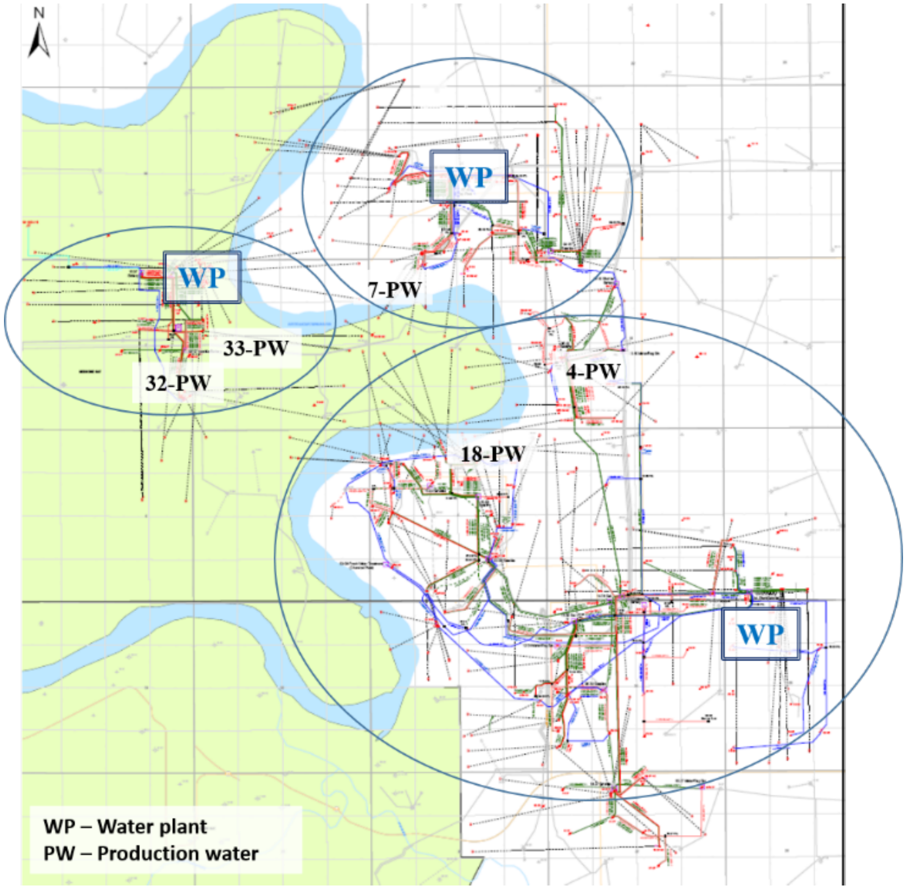

Figure S-1. Production water (PW) wells sampled from the Medicine Hat Glauconitic C (MHGC) field in May, 2015. The corresponding water plant (WP) for each production well surveyed (circled) are also included. Figure adapted from Voordouw et al. (2009).

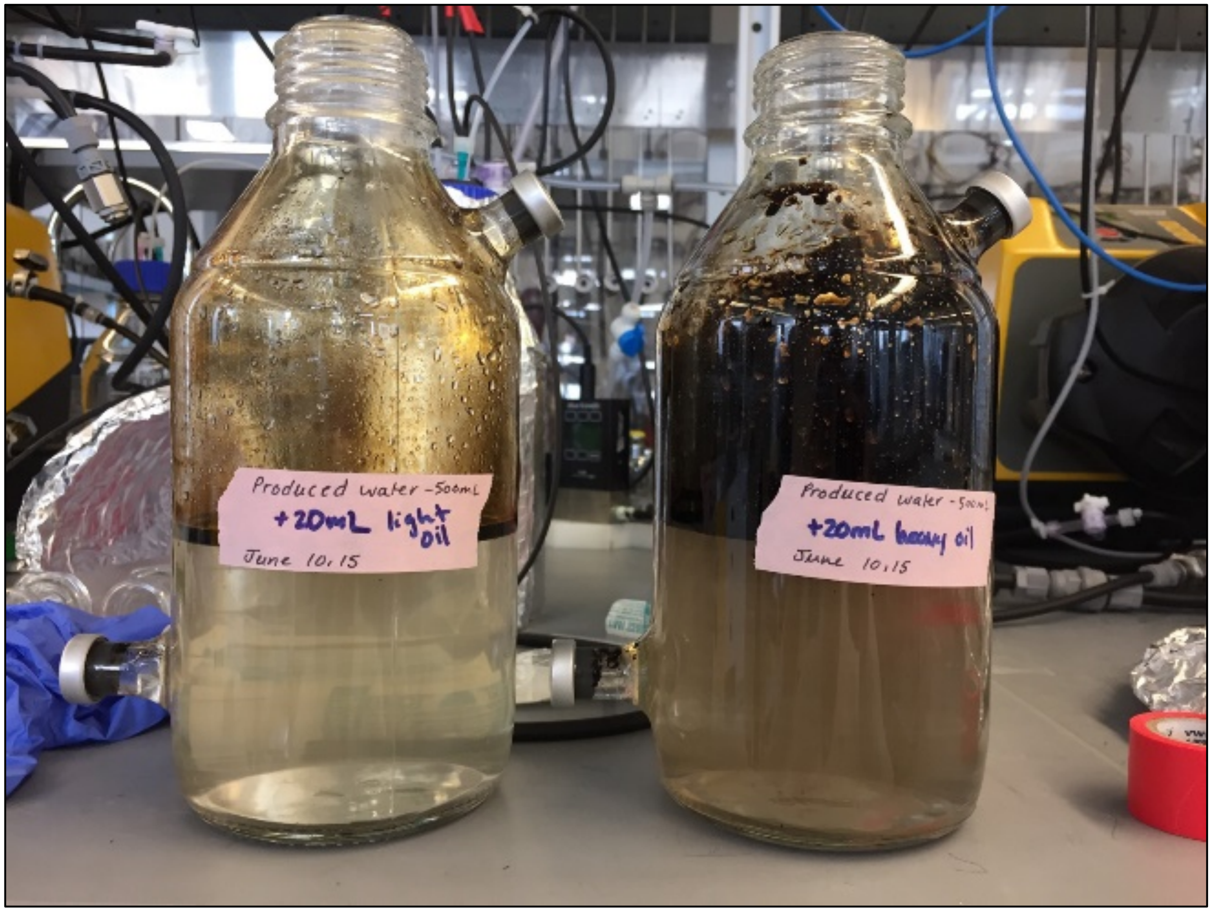

Figure S-2. Image of the light (left) and heavy (right) oil cultures prepared in modified 1-L serum bottles.

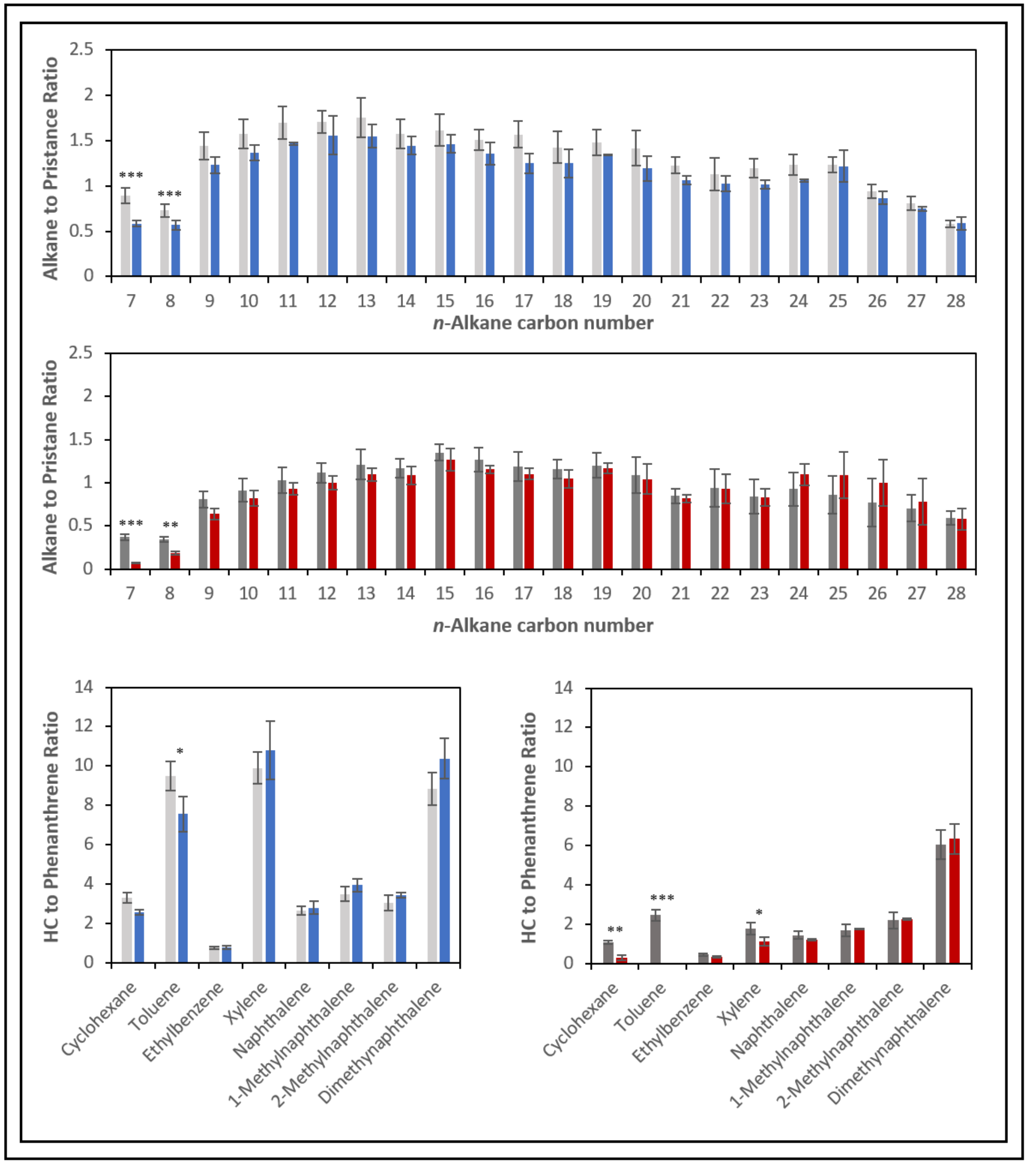

Figure S-3. Hydrocarbon loss in light (blue) and heavy (red) crude oil-amended enrichment cultures relative to corresponding to sterile controls (gray). Hydrocarbon loss was determined as a function of *n*-alkane or hydrocarbon (HC) to pristane or phenanthrene peak area ratios, respectively. Unpaired t-tests were used to determine significance of hydrocarbon loss relative to sterile controls using unpaired *t*-tests; *P* values ≤ 0.05 (*), ≤ 0.01 (**), and ≤ 0.001 (***). Error bars indicate standard deviation of triplicate oil measurements.

Table S-1. Time-resolved quantification of alkane and aromatic hydrocarbon metabolites detected in crude oil-amended cultures. Characteristic ion fragments m/z 262 and $(M - 15)^+$ were selected to probe and integrate TMS-derivatized alkylsuccinates and organic components, respectively. Identification and quantification of metabolites was performed using calibration curves prepared from authentic standards. Color intensity indicates the relative concentration of each metabolite across both cultures over time.

Metabolite	Characteristic ion fragment	Metabolite concentration (μM)												
			**Light Oil**						**Heavy Oil**					
		**T_0**	**T_1**	**T_2**	**T_4**	**T_8**	**T_{12}**	**T_{17}**	**T_1**	**T_2**	**T_4**	**T_8**	**T_{12}**	**T_{17}**
Putative alkylsuccinates from alkanes or cyclic alkanes, signature anaerobic														
**C_1**	262	0.00	0.00	0.12	0.09	0.00	0.00	0.03	0.00	0.00	0.00	0.00	0.00	0.03
**C_2**	262	0.00	0.00	0.19	0.14	0.00	0.00	0.08	0.00	0.07	0.07	0.00	0.00	0.03
**C_3**	262	0.00	0.00	0.44	0.12	0.00	0.00	0.00	0.00	0.00	0.00	0.00	0.00	0.00
***n*-C_3**	262	0.00	0.01	0.25	0.09	0.00	0.00	0.00	0.00	0.00	0.00	0.00	0.00	0.01
**C_4**	262	0.00	0.00	0.09	0.06	0.00	0.00	0.08	0.00	0.00	0.00	0.00	0.00	0.00
***n*-C_4**	262	0.00	0.00	0.18	0.07	0.00	0.00	0.00	0.00	0.00	0.00	0.00	0.00	0.00
**C_5**	262	0.00	0.00	0.64	0.49	0.00	0.00	0.26	0.00	0.43	0.29	0.00	0.00	0.04
**C_5 (w/ unsaturation)**	262	0.00	0.00	1.12	1.08	0.00	0.00	0.81	0.02	1.06	0.85	0.00	0.00	0.16
**C_6**	262	0.00	0.02	1.38	1.45	0.00	0.00	0.83	0.01	1.22	0.99	0.00	0.00	0.42
**C_6 (w/ unsaturation)**	262	0.00	0.03	1.76	1.75	0.01	0.00	1.58	0.01	1.69	1.39	0.00	0.00	0.35
**C_7**	262	0.00	0.00	0.32	0.36	0.00	0.00	0.18	0.00	0.36	0.27	0.00	0.00	0.11
**C_7 (w/ unsaturation)**	262	0.00	0.00	0.71	0.89	0.00	0.00	0.61	0.00	0.77	0.60	0.00	0.00	0.46
**C_8**	262	0.00	0.00	0.24	0.31	0.00	0.00	0.17	0.00	0.30	0.23	0.00	0.00	0.15
**C_8 (w/ unsaturation)**	262	0.00	0.01	0.62	0.71	0.00	0.00	1.00	0.00	0.68	0.46	0.00	0.00	0.67
**C_9**	262	0.00	0.00	0.98	0.84	0.01	0.00	0.00	0.01	1.06	0.84	0.00	0.00	0.68
**C_9 (w/ unsaturation)**	262	0.00	0.00	0.15	0.25	0.00	0.00	0.37	0.00	0.20	0.18	0.00	0.00	0.40
Aromatic and other hydrocarbon metabolites, not uniquely anaerobic														
**Benzoic acid**	179	0.87	5.31	105	44.9	2.11	0.00	75.1	4.26	49.1	28.49	1.11	0.59	30.44
***o*-Toluic acid**	193	0.7	0.77	11.5	10.5	0.69	0.61	8.26	0.76	9.44	8.72	0.64	0.63	9.46

***m*-Toluic acid**	193	0.74	0.85	16.7	15.8	0.61	0.61	1.32	0.85	15.3	13.69	0.00	0.69	0.9
***p*-Toluic acid**	193	0.7	0.84	14.3	13.4	0.71	0.00	10.4	0.79	12.4	11.13	0.65	0.66	9.53
***o*-Cresol**	165	0.44	0.57	7.53	2.91	0.45	0.00	6.28	0.45	1.3	3.07	0.42	0.00	0.47
***m*-Cresol**	165	0.42	0.48	4.13	2.13	0.44	0.00	3.25	0.43	0.47	0.54	0.00	0.00	0.47
***p*-Cresol**	165	0.43	0.51	5.28	0.95	0.42	0.00	1.37	0.43	0.6	0.65	0.00	0.00	0.79
**2-Naphthoic acid**	229	1.22	1.41	31.2	30.3	1.14	1.35	22.3	1.36	29.6	25.76	0.00	1.23	12.88
**Succinic acid**	247	0.01	0.01	0.02	0.02	0.01	0.00	0.03	0.00	0.02	0.02	0.00	0.00	0.03
**Cyclohexane carboxylic acid**	185	1.02	1.29	41.4	32.7	0.97	0.00	34.8	1.23	36.2	44.39	0.00	0.00	15.58
